# Normalization of the Immunological Microenvironment and Sustained Minimal Residual Disease Negativity: Do We Need Both for Long-Term Control of Multiple Myeloma?

**DOI:** 10.3390/ijms232415879

**Published:** 2022-12-14

**Authors:** Giuseppe Bertuglia, Lorenzo Cani, Alessandra Larocca, Francesca Gay, Mattia D’Agostino

**Affiliations:** 1 Division of Hematology, Department of Molecular Biotechnology and Health Sciences, University of Torino, 10126 Torino, Italy; 2 Division of Hematology, Azienda Ospedaliero-Universitaria Città della Salute e della Scienza di Torino, 10126 Torino, Italy

**Keywords:** multiple myeloma, immunological microenvironment, minimal residual disease (MRD), long-term disease control

## Abstract

Over the past two decades, the treatment landscape for multiple myeloma (MM) has progressed significantly, with the introduction of several new drug classes that have greatly improved patient outcomes. At present, it is well known how the bone marrow (BM) microenvironment (ME) exerts an immunosuppressive action leading to an exhaustion of the immune system cells and promoting the proliferation and sustenance of tumor plasma cells. Therefore, having drugs that can reconstitute a healthy BM ME can improve results in MM patients. Recent findings clearly demonstrated that achieving minimal residual disease (MRD) negativity and sustaining MRD negativity over time play a pivotal prognostic role. However, despite the achievement of MRD negativity, patients may still relapse. The understanding of immunologic changes in the BM ME during treatment, complemented by a deeper knowledge of plasma cell genomics and biology, will be critical to develop future therapies to sustain MRD negativity over time and possibly achieve an operational cure. In this review, we focus on the components of the BM ME and their role in MM, on the prognostic significance of MRD negativity and, finally, on the relative contribution of tumor plasma cell biology and BM ME to long-term disease control.

## 1. Microenvironment in Multiple Myeloma: Composition and Role

The bone marrow (BM) niche is the primary residence of both healthy and malignant plasma cells (PCs). Multiple myeloma (MM) is caused by a clonal proliferation of malignant PCs that secretes a monoclonal antibody in the blood or urine, eventually leading to organ dysfunction [1]. It is well established that the BM microenvironment (ME) plays an important role in MM cell growth, survival, progression, and resistance to therapy. The BM ME includes a non-cellular compartment formed by extracellular matrix (ECM) proteins (laminin, fibronectin, and collagen) and a cellular compartment consisting of hematopoietic cells and non-hematopoietic cells.

### 1.1. Osteoclasts, Osteoblasts, and Bone Disease

Bone is a dynamic tissue that is continuously being broken down and restructured in response to many factors. Osteoclasts (OCs) are multinucleated cells that produce enzymes responsible for the dissolution and absorption of bone; on the contrary, osteoblasts (OBs) are large mononuclear cells responsible for the synthesis and mineralization of bone. In MM, bone disease, which is the most frequent clinical characteristic at diagnosis (70–80%) [2], is caused by an imbalance between these two types of cells, namely, an increase in the activation of OCs and a reduction in the number of OBs. An important role is played by the molecular axis formed by the receptor activator of nuclear factor κ B (RANK)–RANK ligand (RANKL)–osteoprotegerin (OPG): RANKL, expressed on the surface of OBs and BM stromal cells, interacts with its receptor RANK, expressed on the surface of OC precursor cells, and stimulates OC formation and bone resorption. OPG, a soluble decoy receptor secreted by BM stromal cells and OBs, binds to RANKL and prevents its interaction with RANK and bone reabsorption. In MM, the increase in RANKL expression and the decrease in OPG expression result in bone resorption [3], and the altered ratio RANK:OPG correlates with survival and bone disease [4]. Other molecules that support osteoclastogenesis in MM are macrophage inflammatory protein-1 (MIP-1) [5,6], stromal-derived factor-1 alpha (SDF-1α)/CXCL12 and its receptor CXCR4 [7], and vascular endothelial growth factor (VEGF) [8,9].

On the other side, MM cells interact with OBs and reduce the levels of OPG [10]. Dickkopf-1 (DDK1) and secreted frizzled-related protein-2 (sFRP2) are produced by MM cells and block the WTN signaling pathway and OB generation [11,12,13]. Transforming growth factor-β (TGFβ), interleukin 7 (IL-7), and hepatocyte growth factor (HGF), all produced by MM cells, are also involved in OB differentiation, reducing the levels of Runt-related transcription factor 2 (RUNX2) and bone morphogenetic protein (BMP) [14,15,16].

**Figure 1:**
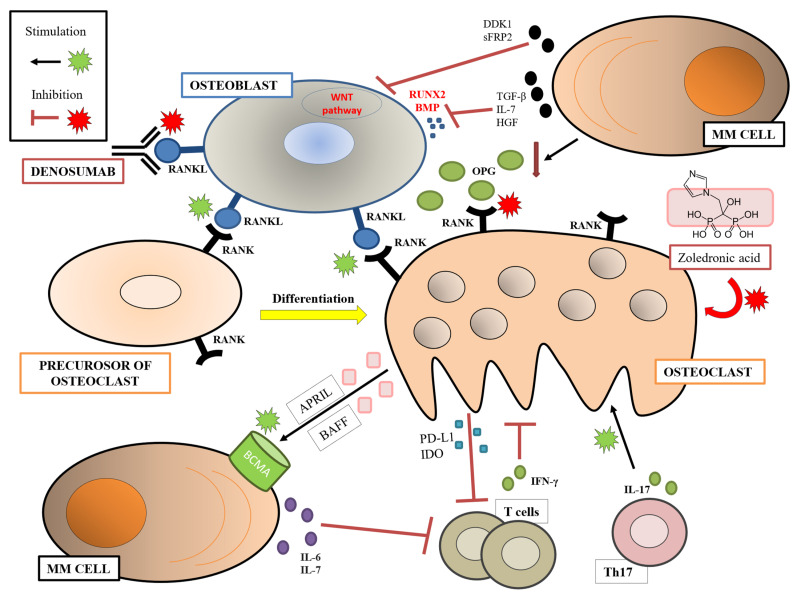
**Schematization of the main factors of bone disease in MM.** RANKL expressed on OBs interact with RANK expressed on the precursors of OCs, resulting in OC differentiation and activation. OPG, a soluble decoy receptor secreted by OBs, binds to RANKL and prevents its interaction with RANK. MM cells interact with OBs and reduce the levels of OPG, preventing the block of osteoclastogenesis. DDK1 and sFRP2, produced by MM cells, block WTN signaling pathway and OB generation. TGFβ, IL-7, and HGF, all produced by MM cells, reduce the levels of RUNX2 and BMP, blocking OB differentiation. Normally, IFN-α produced by T cells strongly suppresses osteoclastogenesis by interfering with the RANKL-RANK axis, but MM cells can induce an up-regulation of RANKL and a down-regulation of IFN-γ secretion by T cells through the mediation of IL-7 and IL-6. Moreover, Th17 lymphocytes stimulate osteoclastogenesis via the production of IL-17. In addition, OCs promote MM survival through the production of APRIL and BAFF and promote immunosuppression through the production of IDO and PD-L1. **Abbreviations:** MM, multiple myeloma; RANK, receptor activator of nuclear factor κ B; RANKL, RANK ligand; OBs, osteoblasts; OCs, osteoclasts; OPG, osteoprotegerin; DDK1, Dickkopf-1; sFRP2, secreted frizzled-related protein-2; WTN, wortmannin; TGFβ, transforming growth factor-β; IL-7, interleukin 7; HGF, hepatocyte growth factor; RUNX2, Runt-related transcription factor 2; BMP, bone morphogenetic protein; IFN-α, interferon-gamma; IL-6, interleukin 6; APRIL, a proliferation inducing ligand; BAFF, B-cell-activating factor; IDO, indoleamine-2,3-dioxygenase; PD-L1, programmed cell death ligand-1.

Furthermore, OCs interact with ME cells and MM cells, contributing not only to the bone disease, but also to immunosuppression and MM cell survival [17,18]. Normally, interferon-gamma (IFN-α) produced by T cells strongly suppresses osteoclastogenesis by interfering with the RANKL-RANK signaling pathway, due to the degradation of tumor necrosis factor (TNF) receptor-associated factor 6 (TRAF6) [19]. MM cells can induce an up-regulation of RANKL and a down-regulation of IFN-γ secretion by T cells through the mediation of IL-7 e IL-6 [20]. In addition, OCs interact with T cells, inhibiting their tumor activity [18]. Conversely, they are the major producer of a proliferation, inducing ligand (APRIL) and B-cell-activating factor (BAFF), which promotes MM survival through the interaction with B-cell maturation antigen (BCMA) and other receptors [21,22] and with immune inhibitor molecules of immune cells, particularly indoleamine-2,3-dioxygenase (IDO) and programmed cell death ligand-1 (PD-L1) [23]. Finally, Th17 lymphocytes seem to stimulate osteoclastogenesis via the production of interleukin-17 (IL-17) [24,25]. The main involved mechanisms are summarized in Figure 1. 

### 1.2. Adipocytes

The bone marrow adipocytes (BMAs) are the most abundant stromal cell phenotype in adult human BM. Adipocyte tissue in BM works not only as a reserve of fatty acids, but also as endocrine organ that secretes adipokines, cytokines, chemokines, and growth factors [26]. Nowadays, the emergent role of BMAs associated with MM cells (MM-BMAs) has become an object of study. MM cells and BMAs metabolically cross talk, since it is demonstrated that MM cells induce lipolysis in adipocytes and then uptake free fatty acids (FFAs) through FA transport proteins (FATPs) 1 and 4, expressed at high levels on the cell surface and within the intracellular space. The expression of FATPs is regulated by FFAs concentration because it stimulates the proliferation of MM cells at lower concentrations and induces lipotoxicity at high concentrations [27]. MM cells can inhibit normal adipogenic differentiation by stimulating the expression of the senescence-associated secretory phenotype (SASP) and other pro-MM molecules, instead of metabolism-related transcripts [28,29]. Indeed, Trotter and colleagues found a significant increase in the number of preadipocytes and mature adipocytes in MM patients, as compared with healthy patients; the adipocyte size was positively correlated with SAPS secretion and lipolysis in adipocytes and, in vitro, with the capacity to promote MM cells migration and proliferation [30].

Furthermore, Liu and colleagues recently reported that MM-BMAs release soluble factors that inhibit osteoblastogenesis and stimulate osteoclastogenesis, such as the increased secretion of DKK1 or PARP family member 9 (PARP9) [31]. Furthermore, MM cells are able to down-regulate the adiponectin secretion of BMAs, at least in part by TNF-α production, setting aside its MM-suppressive effect [32].

### 1.3. Lymphoid Cells and Immune Checkpoint Molecules

In recent years, quantitative and functional alterations have been described in T lymphocytes in patients with MM. Indeed, CD4+ and CD8+ T cells, inversion of the CD4+/CD8+ ratio, abnormal Th1/Th2 CD4+ ratio, and abnormal T cell response have been reported in the literature [33,34]. Zelle-Rieser and colleagues showed that CD8+ T cells have an exhaustive and senescent phenotype in the BM of MM patients and fail to produce IFN-γ [35]. Regulatory T cells (Treg) are a subset of CD4+ T lymphocytes characterized by the surface CD25+CD127low phenotype and the expression of the transcription factor forkhead box P3 (FoxP3) [36]. The increase of Treg in MM patients contributes to the establishment of the immunosuppressive environment. Likely, Treg proliferation and long-term survival depend on elevated APRIL and IDO concentrations, mainly secreted by activated myeloid cells and OCs [37,38]. The developments of Treg and Th17 from naïve T cells are reciprocally interconnected: the presence of the transforming growth factor-beta (TGF-β) alone stimulates the Treg formation, while TGF-β plus IL-6 or IL-21 stimulates the production of Th17 [39]. The elevated concentrations of TGF-β and IL-6 detected in MM patients are probably related to the increased presence of Th17 in this setting of patients. Some studies conducted on a small number of BM samples in MM patients, as compared with those in healthy donors, showed a significant relationship between the proportion of Th17 and clinical states such as the levels of creatine or serum lactate dehydrogenase (LDH) [40,41,42]. In general, MM cells seem to skew the Treg/Th17 balance in favor of a suppressive state [43]. Moreover, as mentioned above, T cells interact with OCs and contribute to bone disease [20,24]. 

Regulatory B cells (Bregs), a small B-cell subset, can regulate immune responses via stimulation of anti-inflammatory cytokine interleukin 10 (IL-10), modulation of CD4+ T-cell activation, and differentiation [44]. Bregs can abrogate NK cell-mediated antibody-dependent cell-mediated cytotoxicity (ADCC) against MM cells. As demonstrated, in the BM of MM patients, Bregs are up-regulated at the time of diagnosis and decrease at the time of response and maintenance therapy [45].

Natural killer (NK) cells play an important role in immune surveillance against viral infections and cancer and are also involved in the MM ME. The studies that analyzed NK cell behavior in MM are controversial: some data revealed a decreased population of NK cells in MM patients [46], while other studies showed an increased number of NK cells in the BM and peripheral blood (PB) of MM patient samples, although with a reduced cytotoxic activity [47,48,49]. In particular, Seymour and colleagues found that NK cells in newly diagnosed (ND)MM exhibit multiple features of NK-cell exhaustion that affect both the more mature CD56dim subset, which normally releases cytotoxic granules, and the more immature CD56bright subset, which releases cytokines [50]. NK cells from MM patients display a reduced expression of activating receptors and a parallel up-regulation of programmed death-1 (PD-1) receptors [51]. 

Immune checkpoints (IC) are positive or negative regulators of the immune system. These pathways are crucial for self-tolerance, which prevents the immune system from attacking cells indiscriminately. Immune tolerance in MM is partly mediated by cytotoxic T-lymphocyte antigen 4 (CTLA-4) and PD-1, two immunomodulatory receptors expressed on T cells that trigger inhibitory pathways dampening T-cell activity. CTLA-4 regulates T-cell proliferation, competing with CD28 for CD80/86 and blocking downstream pathway activation [52]. CTLA-4 is up-regulated in MM T cells and is more prominently expressed in patients with active MM, as compared with monoclonal gammopathy of uncertain significance (MGUS) [53,54]. Similarly, the link between PD-1 on tumor infiltrating T cells and PD-L1 on MM cells inhibits T-cell proliferation. PD-L1 expression levels are higher in MM cells than in cells in MGUS patients and healthy PCs, especially during the relapsed or refractory state [55]. More recently, other immune checkpoint inhibitors are becoming appealing options for the treatment of MM.

T-cell immunoglobulin and ITIM domain (TIGIT) is a coinhibitory receptor that competes with CD226 (DNAM-1) in binding to the ligands CD155 and CD112 on the surface of MM cells [56]. In refractory and progressive MM, it is demonstrated that TIGIT expression is up-regulated on exhausted T cells. This process is at least partially mediated by the IL-10 production via tolerogenic dendritic cells (DCs) [57,58]. 

Lymphocyte activation gene-3 (LAG-3), also called CD223, is a cell surface molecule expressed on activated T cells, NK cells, and B cells that interacts with major histocompatibility complex (MHC)-II to prohibit the binding of the same MHC molecule to T-cell receptor (TCR) and CD4; this results in directly hindering TCR signaling in immune response [59]. Emerging evidence has suggested that MM cells express galectin-3 (GAL-3), which binds to LAG-3, contributing in immunosuppression [60,61]. 

**Figure 2:**
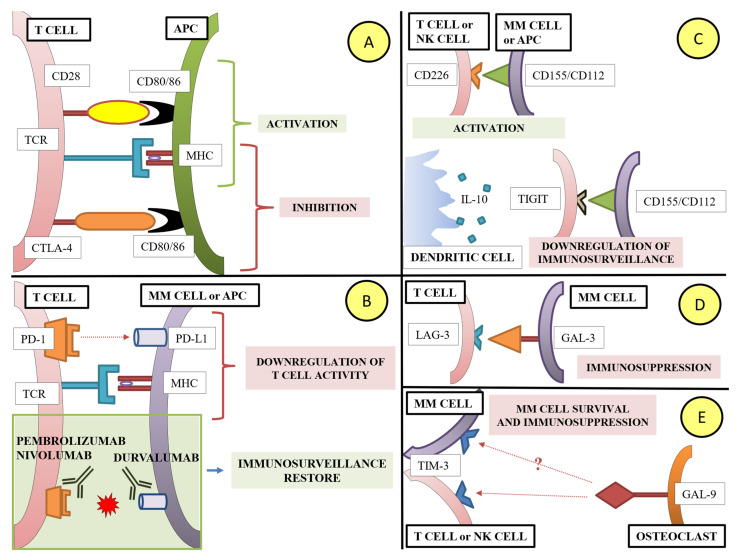
**Main immune checkpoint pathway causing immune escape in MM.** (**Panel A**) The APCs process the antigen, break it into peptides and present it in conjunction with MHC molecules on the cell surface, where it may interact with TCR. In order to proceed with T-cell activation and differentiation, APC must also express co-stimulatory molecules CD80 and CD86, also called B7.1 or B7.2, which are recognized by CD28 expressed on T cells. On the contrary, when CD80/86 binds to CTLA-4, expressed on an exhausted T cell or Tregs, T cells become anergic. (**Panel B**) PD-L1, expressed on APC or MM cell, binds to PD-1 expressed on a T cell, causing down-regulation of T-cell activity. The use of pembrolizumab or nivolumab (anti-PD-1 mAbs) or durvalumab (anti PD-L1 mAb) disrupts the interaction between PD-1 and PD-L1 and can hypothetically restore immune surveillance against MM cells. (**Panel C**) CD226, expressed on T cells, promotes the migration, activation, proliferation, differentiation, and function of CD8+ T cells. The main ligands of CD226 are CD115 and CD112; alternatively, TIGIT is an inhibitory receptor expressed on T cells and interacts with CD155 expressed on APC or tumor cells to down-regulate T-cell and NK-cell functions. IL-10 production by tolerogenic DC seems to have a role in TIGIT expression and exhaustion of T cells. (**Panel D**) LAG-3 is a cell surface molecule, expressed on activated T cells, NK cells, and B cells, that interacts with MHC-II to prohibit T-cell activation. MM cells can express GAL-3, which binds to LAG-3 contributing to immunosuppression. (**Panel E**) TIM-3 expression on T cells or NK membranes is associated with an exhausted phenotype. TIM-3 probably regulates the proliferation of MM cells via the NF-κB signal pathway. GAL-9, expressed by OCs, binds to TIM-3 and participates in inducing immunosuppression. However, the role of GAL-9 on MM cells is not clear. **Abbreviations**. MM, multiple myeloma; APC, antigen presenting cell; TCR, T-cell receptor; MHC, major histocompatibility complex; CTLA-4, cytotoxic T-lymphocyte-associated protein 4; PD-1, programmed death 1; PD-L1, programmed death ligand 1; IL-10, cytokine interleukin 10; TIGIT, T-cell immunoglobulin and ITIM domain; LAG-3, lymphocyte activation gene 3; GAL-9, galectine-9; TIM-3, T-cell immunoglobulin and mucin domain 3; NK, natural killer; DC, dendritic cell; mAbs, monoclonal antibodies; OC, osteoclasts.

The high expression of T-cell immunoglobulin-3 (Tim-3), through the NF-κB signal pathway, negatively regulates NK-cell activity and positively regulates MM-cell survival [62]. CD8+T cells expressing Tim-3 represent a severe decline of its function, and, furthermore, Tim-3+CD8+ T cells usually co-express PD-1 molecules [63,64]. In this scenario, OC-secreted galectin-9 (GAL-9), which can bind to Tim-3, negatively regulates Th1 and NK cells, resulting in the inability to secrete cytokine and reducing the killing activity [17,65,66]. However, the role of GAL-9 in MM is currently controversial because other scholars suggested a pro-apoptotic role of galectin through the up-regulation of c-Jun [67]. The role of IC molecules is summarized in Figure 2. 

Some studies have already investigated the blockade of TIGIT, LAG-3/GAL-3 pathway, resulting in reduction of exhausted T cells, immune activation, and MM-cell cytolysis, and leading to potentially new strategies that could be explored in future clinical trials [57,68,69,70]. 

### 1.4. Myeloid Cells 

As well as lymphoid cells, some myeloid lineages in the BM ME play a key role in MM progression. Macrophages are terminally differentiated myeloid cells, divided into two functional groups: the Th1-derived cytokines promote the “classically activated” or M1 macrophages, which act as antitumoral agents, while the Th2-derived cytokines promote the “alternatively activated” or M2 macrophages, which play an immunosuppressive role that facilitates tumor progression [71]. Tumor-associated macrophages (TAMs), which are included in the second group, are a significant component in the BM of MM patients, constitute around 10% of the BM, and heavily support MM cells proliferation and survival [72]. Indeed, as compared with healthy patients, the infiltration of TAMs in the BM is higher in MM patients, and their numbers further increases in those with an aggressive form of MM [73,74,75]. MM cells influence M2-macrophage polarization, elevating the expression of M2-related scavenger receptor CD206 and blocking LPS-induced TNFα secretion (a hallmark of M1 response) [76].

Moreover, TAMs contribute to angiogenesis through vasculogenic mimicry: MM cells secrete VEGF and FGF-2 that recruit MM-associated macrophages to adapt functionally, phenotypically, and morphologically to endothelial cells and collaborate with them in vessel formation [77,78]. Indeed, TAMS are implicated in the suppression of T-cell proliferation and IFN-γ production [76]. 

On the opposite site, M1 macrophages could induce myeloma tumor cell death by activation of the intrinsic apoptotic pathway [79] and secrete angiostatic chemokines that inhibit vessel proliferation [80]. However, some MM cells are able to modulate antigen secretion, leading to the escape from this immunosurveillance mechanism [81]. 

Myeloid-derived suppressor cells (MDSCs) have been recently identified as immature myeloid cells that are induced by tumor-associated inflammation and are able to impair both innate and adaptive immunity. They are morphologically and phenotypically similar to neutrophils and monocytes, but they potently suppress T cells [82,83,84]. Indeed, their number is significantly higher in patients with MM than in patients with MGUS and healthy controls [85,86,87].

DCs represent a bridge between the innate and adaptative immune responses. Importantly, myeloid DCs (mDCs) and plasmacytoid DCs (pDCs) accumulate in the BM through the MGUS to MM progression. Leone and colleagues showed that DCs play a dual, but opposite, role in MM: they can activate CD8+ T cells against tumor PCs, but they also protect tumor PCs from CD8+ T-cell killing [88]. Indeed, the BM ME impairs DC differentiation, maturation, and activation. TGF-β and IL-10 are both secreted by MM cells and play a significant role in the down-regulation of co-stimulatory molecules CD80/86 during DC maturation; also, vascular endothelial growth factor A (VEGF) is engaged in the impaired DC function due to inhibitory effects on DC maturation and differentiation [89]. 

### 1.5. Influence of Anti-MM Drugs on the ME 

At present, some of the drugs that are widely used in MM care can target the BM ME exclusively. For example, OC-targeting agents such as bisphosphonates and denosumab are approved for the prevention of skeletal-related events (SREs) in MM (see Figure 1). International guidelines recommended initiating bone-targeted therapy concurrently with anti-MM therapy, even in the absence of overt osteolytic lesions [90]. Zoledronic acid inhibits OCs-mediated bone resorption mediated by the inhibition of the enzyme farnesyl pyrophosphate synthase (FPPS), which is an essential enzyme of the mevalonate pathway that leads to a loss of function of the OCs [91]. The Myeloma IX trial demonstrated that zoledronic acid in MM patients had a potential anticancer activity and improved overall survival (OS) independently of the prevention of skeletal-related events (SREs) [92]. Denosumab is a fully human monoclonal antibody (mAb) that inhibits RANKL action [93]. A phase III clinical trial compared denosumab vs. zoledronic acid head to head: the results were similar in terms of SREs and OS, but ad hoc analyses showed an advantage in terms of PFS with denosumab vs. zoledronic acid that was most pronounced among transplant-eligible (TE) patients who received a triplet and/or a proteasome inhibitor (PI)-based induction regimen, thus suggesting a possible synergistic effect [94,95]. 

High-dose chemotherapy (HDC) with melphalan followed by autologous stem-cell transplantation (ASCT) is the current standard of care in patients who are considered TE. The positive results of this approach are due not only to the cytoreductive effect, but to the induction of a pro-inflammatory cytokine burst and disruption of the immune-suppressive tumor ME [96]. The normalization of the BM ME is particularly evident in patients experiencing a very long duration of deep responses after ASCT [97,98]. Similarly to melphalan, cyclophosphamide, another alkylating agent used as debulking therapy or in combination with other drugs, also showed immunomodulatory effects especially on T cells [99]. 

Novel agents, such as PIs, immunomodulatory drugs (IMiDs), and immunotherapies, particularly monoclonal antibodies (mAbs), changed the natural history of MM, determining an important increase in terms of progression-free survival (PFS) and OS. 

**Figure 3:**
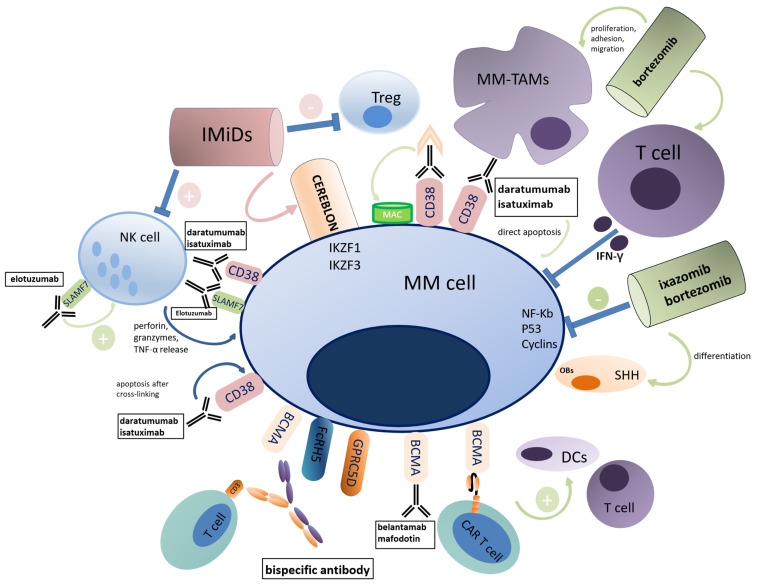
**Schematic representation of anti-MM drugs and their impact on the BM ME.** IMiDs target cereblon on the MM cell surface, promoting the degradation of two transcription factors such as IKFZ1 and IKFZ3. These drugs also have an immune effect, such as the inhibition of Treg cells and the stimulation of NK cells. MAbs can interact with the MM receptor through several mechanisms: the mAb Fc portion binds to the Fc receptor of effector cells, leading to MM cell death by cell lysis (involving NK cells in ADCC); they interact with TAM, promoting the phagocytosis of plasma cells; they induce phospholipidic membrane disruption by involving c1q and the protein cascade with MAC generation. Daratumumab and isatuximab also have a proapoptotic activity, directly or after cross-linking. The BCMA receptor plays a key role in several drug mechanisms. Belantamab mafodotin binds to BCMA and releases monomethyl auristatin F inside the plasma cell. Chimeric antigen receptor (CAR) T cells exert their action through the BCMA receptor and also play a role in the ME by activating DCs and T cells. Bispecific antibodies bind CD-3 on T cells and BCMA/Fcrh5/GPRC5D on MM cells, causing the death of the latter. **Abbreviations**. MM, multiple myeloma; BM ME, bone marrow microenvironment; IMiDs, immunomodulatory drugs; TREG, regulatory T cells; MAC, membrane attack complex; IKZF1, Ikaros family zinc finger 1; IKZF3, Aiolos family zinc finger 3; TAMs, tumor-associated macrophages; IFN-γ, interferon-gamma; NF-kB, nuclear factor kB; OBs, osteoblast cells; SHH, sonic hedgehog pathway; DCs, dendritic cells; CAR T cell, chimeric antigen receptor T cell; BCMA, B-cell maturation antigen; GPRC5D, G protein-coupled receptor family C group 5 member D; FcRH5, Fc receptor-homolog 5; TNF-α; tumor necrosis factor alpha; SLAMF7, signaling lymphocytic activation molecule F7; NK cell, natural killer cell.

In general, all novel agents not only target malignant PCs, but also potentially polarize the immune system from an immunosuppressive state towards cytotoxic behavior (see Figure 3) [100].

The target of the proteasome by PIs (bortezomib, carfilzomib, and ixazomib) interferes with some downstream pathways involved in MM survival (e.g., NF-κB, p53, and cyclins) and it also causes the accumulation of unfolded and misfolded proteins, triggering the apoptosis of PCs [101]. Treatment with bortezomib resulted in a higher recruitment of CD8+ T lymphocytes into the tumor and in higher amounts of tumor-infiltrating IFN-+γT lymphocytes [102]. Bortezomib and zoledronic acid synergistically impact MM-TAM proliferation, adhesion, migration, and cytokine secretion, reducing vasculogenesis as well [103]. Among the mechanisms of resistance to PIs, an increased intracellular ratio between reduced and oxidized glutathione (GSH/GSSG ratio) can allow MM cells to sustain high protein production and proliferation. However, this process is supported and potentially driven by the surrounding ME [104]. 

Similarly to bortezomib, some studies showed that carfilzomib also potently reprogrammed TAMs into M1-like macrophages [105], while ixazomib decreased vasculogenesis and osteoclastogenesis and concomitantly increased OB differentiation, throughout the activation of sonic hedgehog (SHH) signaling pathway [106]. 

IMiDs (thalidomide, lenalidomide, and pomalidomide) are a class of drugs with pleiotropic effects, including immunostimulatory properties on the ME components and direct antitumor activity against MM cells. The primary target is cereblon (CRBN), a ubiquitously expressed protein that forms with other proteins a complex called cullin-4 RING E3 ligase (CRL4), an E3 ubiquitin ligase: IMiDs stabilize CRBN, leading to an enhanced affinity for two zinc finger transcription factors Ikaros (IKZF1) and Aiolos (IKZF3), with subsequent ubiquitination and degradation of these transcription factors [107]. These proteins are overexpressed in MM cells and ensure their proliferation and survival through a mechanism involving the reciprocal stimulation of c-Myc and interferon regulatory factor 4 (IRF4), which are two critical oncogenes [108]. Regarding the immune system, different scholars reported that T cells increase their cytokine production after IMiD exposure [109,110,111,112,113]. In fact, the degradation of Aiolos and Ikaros binds and suppresses the IL-2 promoter region, leading to an increased secretion of proinflammatory IL-2 [114]. Furthermore, lenalidomide and pomalidomide strongly inhibit Treg proliferation and MDSCs [115,116]. IMiDs have also been shown to increase NK cell cytotoxic activity, partly indirectly via the mediation of IL-2 production and partly directly [117,118]. Similarly, IMiDs improve the maturation and functionality of MM patient-derived DCs as antigen-presenting cells, probably through Aiolos and Ikaros pathways [109]. Finally, the release of neoantigens due to MM cell apoptosis and the concomitant reactivation of immune actors could have a synergistic effect in maintaining a durable response [119]. 

MAbs target neoplastic cells and activate the immune system or disrupt a signaling pathway protecting neoplastic cells from immune-cell destruction [120]. In this setting, anti-CD38 are represented by two IgG kappa antibodies, daratumumab and isatuximab. CD38 is a glycoprotein acting as ectoenzyme, which catalyzes the conversion of nicotinamide adenine dinucleotide (NAD) to adenosine diphosphate ribose (ADPR) and nicotinic acid adenine dinucleotide phosphate (NAADP), and is thus involved in the mobilization of intracellular calcium useful in signaling pathways of cell growth, survival, and differentiation [121]. CD38 is expressed on PCs as well as in other hematopoietic and non-hematopoietic cells. Following the binding to CD38, daratumumab exerts its cytotoxic effects through several mechanisms, including complement-dependent cytotoxicity (CDC), antibody-dependent cell-mediated cytotoxicity (ADCC), and antibody-dependent cellular phagocytosis (ADCP), as well as through the direct induction of apoptosis upon secondary cross-linking [122]. This mechanism of action is shared by isatuximab, although the binding epitope is different from the previous one [123].

CD38-expressing Tregs (which are a subpopulation more immunosuppressive in vitro than CD38-negative Tregs, Bregs, and MDSC) are sensitive and decrease in number after daratumumab treatment. Moreover, the number of cytotoxic T cells increases after daratumumab treatment, determining increased CD8:CD4 and CD8:Treg ratios [124]. These changes are more evident in responding patients than in non-responders [125]. CD38+ NK cells decline after daratumumab exposure in PB and BM, although the remaining NK cells may still contribute to ADCC, clinical efficacy, and infection control [126,127]. The same was demonstrated after isatuximab exposure [128]. Casneuf and colleagues analyzed daratumumab effects in combination with lenalidomide and dexamethasone (Rd) vs. lenalidomide and dexamethasone in the POLLUX trial, showing that daratumumab in combination with Rd induced deeper effects in NK cells and CD8+ activation than Rd alone, especially in deep responders [129,130]. Moreover, CD38 could work as a sensor able to regulate OCs, its Ca^2+^ signaling pathway; thus, anti-CD38 therapy could mitigate bone disease either by restoring T-cell function or by inhibiting early osteoclastogenesis [131,132,133]. Unfortunately, MM cells can develop several mechanisms of resistance to anti-CD-38 therapy, primarily CD38 down-regulation, depletion of NK cells via fratricide ADCC against nearby NK cells (as previously described), and immune escape of MM cells through the inhibition of ADCP by overexpression of the CD47 “don’t eat me” signal [134,135]. However, the combination of daratumumab with IMiDs could partly overcome the refractory status due to the enhancement of immune stimulation [136]. 

Elotuzumab, an immunostimulatory mAb targeting signaling lymphocytic activation molecule F7 (SLAMF7), did not show an effective anti-MM activity as single agent, but showed activity in combination with lenalidomide and pomalidomide in patients with relapsed/refractory (RR)MM, even if it failed in the first-line setting [137,138,139]. This mAb can facilitate NK cell-mediated ADCC of MM cells through Fc-dependent interactions with FcγRIIIA (CD16), it can promote macrophage-mediated ADCP of MM cells through Fc-dependent interactions with Fcγ receptor, and it can also directly bind to SLAMF7 on NK cells and activate them [140,141]. Furthermore, Awwad and colleagues recently found that SLAMF7 is a highly expressed marker on the surface of suppressive CD8+ T cells and that its expression correlates with an exhausted phenotype in T cells. Thus, SLAMF7+ CD8+ Treg cells could be eliminated via ADCC, contributing to reduce immunosuppression [142]. Finally, elotuzumab seems not only to spare the function of DCs, but also to enhance the IL-2 immune response of DCs induced by IMiDs [143,144]. 

Although immune exhaustion plays an important role in the pathogenesis of MM, at present, there are no available drugs targeting immune checkpoint (IC) molecules (see Figure 2). Two phase III trials, KEYNOTE-185 (NCT02579863) and KEYNOTE-183 (NCT02576977), respectively investigated pembrolizumab (an anti-PD-1 mAb) in combination with Rd vs. Rd alone in transplant-ineligible (NTE) NDMM patients and pembrolizumab plus pomalidomide-dexamethasone (Pd) vs. Pd alone in RRMM patients. In both trials, potentially better results were observed in association with IMiDs, probably due to the possible synergistic effect on the immune system. Nevertheless, the toxicity reported led the Food and Drug Administration (FDA) to halt trials exploring these combinations [145,146,147,148,149]. As described above, other ongoing trials are investigating the role of other IC inhibitors in MM [150].

BCMA is a new target in MM therapy. This protein is expressed on the surface of normal late mature B-lymphocytes and is overexpressed in MM cells. The interaction of APRIL and BAFF with BCMA results in the proliferation, differentiation, and survival signal of MM cells [151].

Belantamab mafodotin is the first approved antibody–drug conjugate (ADC) containing an IgG anti-BCMA mAb conjugated with monomethyl auristatin F (MMAF), a microtubule inhibitor, thanks to the results of the DREAMM-1 and DREAM-2 clinical trials [152,153]. In addition to the direct death of MM cells, anti-BCMA probably interfere with the symbiosis between MM cells and OCs, blocking the sustenance of MM cells mediated by the interaction between BCMA and APRIL, the latter expressed by OCs [21,22]. BCMA can also be shed from the surface of PCs (more specifically, by the γ-secretase) and can circulate in the serum in a soluble form (sBCMA), sequestering the circulating BAFF and preventing its normal stimulation of normal B cells [154,155]. Thus, the combination of belantamab mafodotin and γ-secretase inhibitor (GSI) could have a synergistic effect, avoiding the resistance to belantamab mafodotin for the loss of BCMA on the surface of MM cells, and it is being investigated in the DREAMM-5 clinical trial (NCT04126200) [156]. 

Immunotherapies based on chimeric antigen receptor (CAR) T cells, which are T cells engineered with a particular T-cell receptor, showed an impressive overall response rate (ORR) in several clinical trials [157,158,159]. A study by Dhodapkar et al. on the BM ME pre- and post-BCMA-specific CAR T-cell therapy showed that the duration of response may depend on the dynamic interplay between endogenous T cells, CAR T cells, and DCs: the proportion of BM T cells and DCs increased after treatment, while suppressor myeloid cells decreased. Importantly, these changes were not observed in patients with a short PFS [160]. Indeed, some patients relapsed with BCMA+ MM cells, while circulating anti-BCMA CAR T cells were still being detected, suggesting that CAR T-cell persistence and antigen expression on target cells may be necessary but not sufficient to exert long-lasting antitumor immunity [161]. 

The clinical success of anti-BCMA CAR T-cell therapy prompted the further development of different T-cell-directing immunotherapies: bispecific antibodies (bsAbs) or bispecific T-cell engagers (BiTEs^®^). Specifically, bsAbs contain the Fc domain and induce additional immune responses mediated by innate immune cells and/or the complement system, while BiTEs^®^ do not contain the Fc region and consist of two different single-chain variable regions [162]. 

Teclistamab is a T-cell-redirecting bsAb that targets both CD3 expressed on T cells and BCMA expressed on MM cells and it is the first bsAb drug approved by the European Medicines Agency in the RRMM setting, due to the promising results of the ongoing phase I/II MajesTEC-1 study (NCT04557098) [163]. Subsequent studies showed that BM components (e.g., a high percentage of Tregs) could reduce the rate of response, suppressing the proliferation of effector T cells previously activated by bsAbs. In this setting, Meermeier and colleagues found that, by reducing tumor burden and depleting regulatory T cells, cyclophosphamide prevented bsAb-induced T-cell exhaustion and promoted long-term MM control [164]. Talquetamab is a bsAb that targets CD3 and G-protein coupled receptor family C group 5 member D (GPRC5D), a recently identified MM antigen that is highly expressed on malignant MM cells and lowly expressed on hair follicles, but not on other healthy cells. The FDA has recently granted breakthrough therapy designation to talquetamab for the treatment of RRMM, based on the positive results from the phase 1/2 MonumenTAL-1 study [165]. Another phase I clinical trial is investigating, with promising results, the role of cevostamab, an IgG-based T-cell-engager bsAb directed to CD3 and Fc receptor-homolog 5 (FcRH5), a membrane protein selectively expressed on B cells and PCs [166]. With several phase I clinical trials investigating MM-targeting bsAbs currently underway and with the possibility to use them in combination with the already known drugs, the scenario could remarkably change in the next years. 

The effects of anti-MM drugs on the BM ME are summarized in Table 1.

In this scenario, more efforts have been made to directly target the BM ME components, for instance, the MM-associated macrophages (M1) in order to activate their antitumor activity against MM cells [173]. Nevertheless, some clinical trials targeting the BM ME have failed due to toxicity or unsatisfactory results. Selected clinical trials are summarized in Table 2.

## 2. MRD in MM: Relevance as Prognostic Factor

In the last decade, new therapeutic options for MM have substantially progressed, making possible the achievement of a complete response (CR) after the first line of therapy in about 50% of patients eligible and not eligible for ASCT [178,179,180,181].

In many studies, the achievement of a CR (defined by immunofixation-negative blood and urine and <5% of PCs in the BM) was correlated with better PFS and OS rates [182]. However, recent data showed that achieving a CR with the persistence of minimal residual disease (MRD) did not offer a better prognosis in terms of PFS and OS, as compared with the achievement of responses such as a very good partial response (VGPR) or a partial response (PR) [183,184]. It is definitely clear that the real prognostic significance of CR is tied to the MRD status, and because of this evidence, MRD was included by the International Myeloma Working Group (IMWG) in the response criteria. Furthermore, it was also incorporated as secondary endpoint and, more recently, even as primary endpoint in several clinical trials [185].

Inside the BM, techniques for MRD evaluation such as next-generation sequencing (NGS) and multiparameter flow cytometry (MFC) are actually standardized and recommended by the IMWG updated response criteria [185], while, at the extramedullary level, the main radiological technique is the positron emission tomography/computed tomography (PET/CT).

MFC analyzes the expression of surface antigens that are typical of PCs (CD138 and CD38) or aberrant markers (CD20, CD56, CD19, CD45, CD27, CD28, CD33, and CD117) and analyzes the monoclonal expression of intra-cytoplasmic markers (intracellular κ or λ chains). Currently, next-generation flow (NGF) is the most used type of MFC in clinical trials, given its higher sensitivity (up to 10^−6^) and more consistent reproducibility, as compared with the standard 8-color MFC [186,187]. 

NGS detects the malignant clone by sequencing VDJ and DJ rearrangements on immunoglobulin chain genes, with a maximum sensitivity of 10^−6^. Sensitivity is high for both NGF and NGS, and they also have a good concordance level (80–85%) that changes according to the cut-off chosen for MRD detection [188,189]. In particular, data from the FORTE trial showed that the outcomes in patients who were MRD negative by MFC at a sensitivity of 10^−5^ and by NGS at 10^−5^ were similar, confirming the high concordance between the two techniques [187].

A large meta-analysis revealed that achieving MRD negativity reduced of about 50% the risk of progression and mortality [183]. Despite this, a significant percentage of these patients relapsed and died from the disease [183]. This can be explained by the poor sensitivity of the older MRD techniques used in this meta-analysis, such as the first-generation 4-color MFC.

So, what is the best sensitivity cut-off for achieving clinically relevant results with this technique? Data regarding NGS [190] showed that a deeper response can offer better outcomes. For example, an MRD level below 10^−6^ was predictive of a higher PFS, as compared with 10^−5^ or 10^−4^. Therefore, a cut-off of 10^−6^ turns out to be a strong prognostic biomarker of PFS and OS [191].

As discussed, a sensitivity of 10^−6^ can also be achieved by NGF as well. Thus, it is important to note that the methodology used is not as important as long as deeper levels of MRD can be measured [185]. 

It is clear that achieving MRD negativity represents one of the most important therapeutic aims in fit NDMM patients. With the most effective combinations, MRD negativity can be reached in up to 80% of NDMM patients [192], although sustaining MRD negativity over time can still be challenging. The optimal duration of MRD negativity is still an open issue. San-Miguel and colleagues evaluated sustained MRD negativity lasting ≥6 or ≥12 months, in patients with NDMM not eligible for transplantation in the MAIA and ALCYONE studies. They confirmed the positive impact of achieving MRD negativity on PFS and, furthermore, demonstrated how this benefit was higher in patients sustaining MRD negativity for at least 12 months [192].

In addition, MRD negativity compared with MRD positivity had longer times to subsequent anticancer therapy, showing that the residual MM clone in the BM predicted a need for treatment during the subsequent follow-up [192]. 

MRD negativity and duration of MRD negativity were also measured in the FORTE trial in patients with TE NDMM, showing that the treatment arms with the highest rates of sustained MRD negativity predicted the best outcomes in terms of PFS [193]. 

Nevertheless, even in case of sustained MRD negativity at high sensitivity, relapse may still occur. Some factors—such as the malignant PC biology and the immunological state of the BM ME—may still play a prognostic role in the context of MRD negativity after initial treatment.

## 3. Prognostic Impact of PC Biology on Sustained MRD Negativity

At present, there is no personalized treatment for patients who achieve MRD negativity. Several trials are underway, investigating the possibility to de-intensify or even discontinue MM therapy in patients who achieved sustained MRD negativity. The hypothesis of this MRD-driven action is that the outcome of the patient should not be impacted by treatment if sustained MRD negativity is reached. In the phase II, single-arm MASTER trial, patients were treated with daratumumab, carfilzomib, lenalidomide, and dexamethasone (Dara-KRd) followed by ASCT, and MRD guided post-ASCT intensification or cessation of therapy. MRD was assessed at the completion of induction, 60–80 days after ASCT, and after the second cycle of Dara-KRd in each phase of consolidation (i.e., cycles 6 and 10). Patients reaching two consecutive MRD-negative assessments at 10^−5^ transitioned to treatment-free observation at the end of the corresponding phase. Eighty-four patients (71%) achieved two consecutive MRD assessments at 10^−5^.

In this subset, patients with two or more high-risk cytogenic abnormalities (HRCA; defined by two or more alterations including t(4;14), t(14;16), del(17p), gain or amplification of 1q, and t(14;20)) had a higher rate of MRD resurgence at 12 months than patients with just one or zero HRCA (27% vs. 0% and 4%, respectively).

The findings of this study showed the opportunity for a de-escalation of therapy in patients who achieved a deep response. On the other hand, it showed how different approaches may be needed in very-high-risk patients [194].

Analyses on predictors of unsustained MRD patients were also conducted in the FORTE trial. Despite the achievement of MRD negativity, high levels of circulating tumor plasma cells (CTC), amp(1q), and the co-occurrence of multiple HRCA identified a population of patients at higher risk of losing their MRD-negative status over time. However, they also found that maintenance therapy with two drugs (carfilzomib and lenalidomide) rather than one (lenalidomide) significantly reduced the risk of MRD re-positivization [195].

In addition to these findings, Goicoechea et al. analyzed data from the patient pool of the phase III study PETHEMA/GEM2012-MENOS65, observing that the depth of response of MRD remained lower in high-risk MM patients despite intensive therapy. However, the rate of sustained MRD negativity from induction to consolidation was similar between patients with or without HRCA, and the PFS rate at 36 months was greater than 90% in patients with undetectable MRD, with no significant differences between patients with or without HRCA. In addition, no significant differences were found in the outcomes of patients with undetectable MRD when stratified according to the presence of t(4;14), t(14;16), and del(17p13) (36-month PFS rates of 100%, 100%, and 89%, respectively). Taken together, these results suggested that achieving sustained MRD negativity could overcome the poor prognosis of patients with HRCA [196].

The poorer outcomes conferred by the high-risk alterations compared with the standard ones in patients with similar MRD log levels raised interest in the patterns of mechanism of resistance in the residual cells of two subgroups. Interestingly, higher genomic instability and acquisition of new mutations in residual tumor cells was detected in patients with HRCA than in standard-risk patients; in the latter, most mutations and copy-number alterations present at diagnosis were not found in residual tumor cells. This finding revealed greater clonal selection in standard-risk MM, whereas greater genomic instability would lead to the acquisition of new mutations in MRD cells of patients with high-risk cytogenetic abnormalities.

Goicoechea and colleagues also found a mechanism of resistance in the MM high-risk residual cells of patients resistant to bortezomib. These cells showed an up-regulation in antioxidant circuits, which would lead to a reduction in reactive oxygen species (ROS)-mediated cytotoxicity and subsequent enhanced survival [196]. 

All of these findings show how a deeper response to therapy can actually be modulated by the genomic and metabolic changes of the plasma cell, which undergoes the pressure of therapy and can be affected by the changes that occur within the ME.

## 4. Prognostic Impact of the BM ME on Sustained MRD Negativity

Many studies are currently investigating how the BM ME can influence the maintenance of a deep response, identifying immune profiles that would find subsets of patients that are prognostically more or less favorable to response to therapy.

Paiva and colleagues demonstrated that offering complementary flow-based information to the quantification of MRD levels by immune profiling of the ME in NTE patients was prognostically relevant, identifying a subset of patients who, albeit being MRD positive, could still experience prolonged survival due to a unique immune signature specifically characterized by a more prominent regeneration of mature B lymphocytes [197]. Barlogie and colleagues demonstrated that a similar immune signature was previously found in both MRD-negative and MRD-positive MM patients reaching long-term disease control [198]. It was demonstrated that patients who achieved gradual and sustained MRD negativity during lenalidomide maintenance reached a gradual normalization of the immune ME, while patients with MRD positivity had an immune ME permanently dysregulated [199]. 

In the MASTER trial, Gowda and colleagues further explored the immune reconstitution (IR; characterizing quantitative changes in the repertoire of immunoglobulin genes by NGS and serum gamma globulin levels) in patients undergoing autologous hematopoietic cell transplantation (AHCT) and eventual MRD-guided consolidation. They found a delayed IR in patients who received post-AHCT consolidation compared with those who did not, a rapid expansion in the immunoglobulin repertoire with a plateau at 6 months for both groups, and no difference 18 months after treatment cessation [200]. It is still unknown if this is important only regarding anti-infection immunity or also regarding anti-tumor immunity.

Another approach is to correlate ME findings with tumor characteristics. Maura and colleagues found that distinct genomic lesions were associated with distinct immune-ME composition: del(22q):XBP1 was associated with fewer memory B cells, fewer naïve B cells, and fewer DCs, while del(6p) was associated with fewer T cells CD8+. Furthermore, the gene expression and ME composition were different in responding and non-responding patients: inflammatory response genes (i.e., IL1B expression) were characteristic of persistently MRD-positive patients, whereas genes implicated in IL2, IL6, and IFN-α response as well as in adipocyte differentiation were associated with sustained MRD response [201]. 

## 5. Discussion

Despite the improved therapeutic scenario in the treatment of MM, the survival of certain patients remains poor in the clinical trial as well as in the real-world settings [202]. Up to now, MM therapy has been tailored only based on transplant eligibility. Very few actions are recommended according to risk, such as the possibility of performing a tandem HDC-ASCT in high-risk patients [203] and the possibility of using the BCL-2 selective inhibitor venetoclax in patients with t(11;14) [204]. Nevertheless, MM is a heterogeneous disease whose aggressiveness depends on several variables, such as the tumor burden, the cytogenetic risk of the PCs, and the composition and influence of the BM ME. 

Over the years, various prognostic tools to stratify NDMM patients into different risk groups have been proposed, not all of them easily applicable in the real-world setting [205]. There is a growing evidence suggesting that disease risk is dynamic and that the achievement of MRD can modify/decrease baseline risk and in some cases abrogate it, especially with the occurrence of sustained MRD negativity [206].

Most ongoing clinical trials are exploring active areas of research. MRD assessment at predefined time points (after induction, after HDC/ASCT, after consolidation, and during maintenance) helps understand the role of MRD-driven therapy, allowing for intensification or deintensification of treatment. Besides, cytogenetic risk and patient fitness are other factors to be considered. Some examples of this strategy include the evaluation of a second ASCT after the first one, the need for post-transplant consolidation therapy, the duration of maintenance therapy, or the possibility of discontinuing therapy in NTE patients by reducing toxicity [206]. Other ongoing clinical trials are investigating the use of drugs against other ME components as main targets; these include tumor (T)ME physical barriers (extracellular matrix, fibroblast activating protein collagen, and laminin), immune checkpoints such as CTLA-4 and PD-1/PD-L1, MDSCs, immunosuppressive macrophages, Tregs, inhibitory cytokines, and metabolic inhibition signaling [207]. If these strategies will be effective and safe, their use in combination with other drugs already used in MM therapy will change and improve the survival of MM patients. 

Even if undetectable MRD seems to overcome the dismal survival of patients with high-risk MM (even of patients with sustained MRD negativity), the MASTER trial demonstrated that multiple HRCA negatively influenced the outcome of these patients, suggesting that discontinuing therapy may not be the best option in these patients [183]. However, in the FORTE trial, patients with multiple HRCA, 1q amplification, and high baseline levels of CTC showed a high rate of MRD reappearance despite continuous treatment. In this context, the use of a 2-drug maintenance treatment mitigated the risk of unsustained MRD negativity, as compared with a 1-drug maintenance approach [195].

Guerrero and colleagues put together PC biology factors and ME factors in a machine learning algorithm to predict MRD outcomes and, consequently, better or worse outcomes. They found that the most effective model to predict MRD status (after bortezomib-Rd [VRd] induction, HDT/ASCT, and VRd consolidation) resulted from integrating cytogenetics, tumor burden (BM PC clonality detected by MFC and CTC), immune-related biomarkers of myeloid precursors, neutrophils, eosinophils, B cells, T cells, and NK cells [208,209]. The performance of the machine learning model integrating tumor and ME factors in predicting PFS was superior to that observed using the Revised International Staging System (R-ISS). 

The ability of predicting patients who have more chances to achieve sustained MRD negativity can be a guide to define personalized therapy strategies. The development of a more accurate algorithm to analyze a large number of variables could make this possibility a reality in the near future.

New predictive factors were also analyzed by Tahri and colleagues, who investigated how the presence of specific subsets of NK cells in the BM ME could give worse outcomes in NDMM patients undergoing first-line therapy with daratumumab. They observed that, in a cohort of frail NDMM patients ineligible for transplantation, about 20% of them showed a reduced number of cytotoxic NK cells. This was correlated with a shorter PFS after a first-line therapeutic regimen including the anti-CD38 daratumumab [210]. These results suggested how the accurate characterization of the BM ME and, in particular, of the type of NK cells, could be useful to stratify groups of patients who could benefit less from therapies relying on NK-cell mediated ADCC.

## 6. Conclusions

Many drugs for the treatment of MM have recently been approved. Nonetheless, the fight against this disease is still difficult due to its complex biology and the various parameters that influence the outcome of patients. Improving the ability to predict their future outcome remains paramount. Despite the availability of tools to evaluate disease risk at baseline and the possibility to monitor MRD after treatment, some patients still have an unpredictable disease course. Genomic characterization of the BM ME and PC biology should both be considered to better define high-risk patients and to design risk- and response-adapted strategies.

## Figures and Tables

**Table 1:** **Effects of anti-MM drugs on the BM ME**.

Drug Group	Drug Name	Drug Mechanism			Effect on the ME
Bisphosphonates	Zoledronic acid	Inhibition of FPPS			- Inhibition of OC-mediated bone resorption [91] - Proliferation of M1 phenotype TAMs [103]
Alkylating agents	Melphalan	Alkylation of DNA at the N7 position of guanine and induction of DNA inter-strand cross-linkages			- Leucodepletion [96] - Myelodepletion - Activation of CD8+ T cells [96]
	Cyclophosphamide	DNA damage (DNA strand breaks)			- Polarization of Th1 [99,167] - Depletion of Tregs [99,167] - Activation of DCs [99,168] - Polarization of the M1 phenotype TAMs and enhancement of ADCP [99]
Proteasome inhibitors (PIs)	Bortezomib	It binds reversibly to the β 5 subunit of the proteasome [169]	Inhibition of NF-κB pathway [101] SHH signaling pathway activation *		- Recruitment of CD8+-T lymphocytes and IFN-γ+ lymphocytes [103] - Proliferation of M1 phenotype TAMs [103]
	Carfilzomib	It binds irreversibly to the β 5 subunit of the proteasome [169]			- Reprogramming of TAMs into M1-like macrophages [105]
	Ixazomib	It binds reversibly to β 5 subunit and β at high concentration of the proteasome [169]			- Decrease in vasculogenesis [106] - Inhibition of OC-mediated bone resorption [106] - Induction of OB activity [106]
Immunomodulatory drugs (IMiDs)	Thalidomide	Degradation of transcription factors Ikaros and Aiolos through cereblon stabilization [170]			- Induction of CD8+ T-cell cytotoxicity [171] - Increase in NK-cell cytotoxicity [116]
	Lenalidomide				- Induction of CD8+ T-cell cytotoxicity [171] - Inhibition of Treg proliferation [114] - Inhibition of MDSCs [115] - Induction of DC activation [109]
	Pomalidomide				- Induction of CD8+ T-cell cytotoxicity [171] - Inhibition of Treg proliferation [114] - Inhibition of MDSCs [115] - Induction of DC activation [109]
Monoclonal antibodies (mAbs)	Denosumab	Anti-RANKL [93]			- Inhibition of OC activity [94,95]
	Daratumumab	Anti-CD38		ADCC ADCP CDC [123]	- Inhibition of CD38+ Tregs [124] - Inhibition of CD38+ Bregs [124] - Inhibition of CD38+ MDSCs [124] - Reduction of CD38+ NK cells [124,126,127] - Increase in CD8+ T-cell cytotoxicity [124] - Inhibition of osteoclastogenesis [131,132]
	Isatuximab	Anti-CD38		ADCC ADCP CDC [124]	- Inhibition of CD38+ Tregs [128] - Inhibition of CD38+ Bregs [128] - Inhibition of CD38+ MDSCs [128] - Reduction of CD38+ NK cells [128] - Increase in CD8+ T-cell cytotoxicity [128]
	Elotuzumab	Anti-SLAMF7		ADCC ADCP [172]	- Activation of NK cells [141] - Reduction of SLAMF7+ Treg cells [142] - Enhancement of DC activity [143,144]
	Pembrolizumab	Anti-PD-1			- Activation of Th1 and their cytokine secretion [149] - Inhibition and killing of Tregs [149]
Antibody–drug conjugates (ADC)	Belantamab mafodotin	Anti-BCMA combined with mafodotin (tubulin inhibitor)			- Disruption of the interaction between the BM ME and MM cells, depending on BCMA [21]
Chimeric antigen receptor (CAR) T cells	- Idecabtagene vicleucel (ide-cel) - Ciltacabtagene autoleucel (Cilta-cel)	Anti-BCMA			- Activation of cytotoxic T cells [160] - Activation of DCs [160] - Reduction of MDSCs [160]
Bispecific antibodies (bsAbs)	Teclistamab	Anti-BCMA and CD3			- Activation of CD3+CD8+ T cells [162]
	Talquetamab	Anti-GPRC5D and CD3			- Activation of CD3+CD8+ T cells [162]
	Cevostamab	Anti-FcRH5 and CD3			- Activation of CD3+CD8+ T cells [162]

* The blockade of SHH signaling is mediated only by ixazomib. **Abbreviations**: MM, multiple myeloma; BM ME, bone marrow microenvironment; FPPS, farnesyl pyrophosphate synthase; NF-κB, nuclear factor kappa-light-chain-enhancer of activated B cells; SHH, sonic hedgehog; RANKL, receptor activator of nuclear factor κ B; ADCC, antibody-dependent cellular cytotoxicity; ADCP, antibody-dependent cellular phagocytosis; CDC, complement-dependent cytotoxicity; PD-1, programmed death-1; BCMA, BCMA, B-cell maturation antigen; GPRC5D, G protein-coupled receptor family C group 5 member D, ME, microenvironment; OCs, osteoclasts; TAMs, tumor-associated macrophages; DCs, dendritic cells; IFN-α, interferon-gamma; OBs, osteoblasts; NK, natural killer; MDSCs, myeloid-derived suppressor cells; Tregs, regulatory T cells; SLAMF7, signaling lymphocytic activation molecule F7.

**Table 2:** **Ongoing or terminated clinical trials including drugs targeting the BM ME**.

Drug	Drug Class and Mechanism	ClinicalTrials.gov Identifier	Study Phase	Description	Status	Results
BI-505	MAb directed against intercellular adhesion molecule 1 (ICAM-1)	NCT02756728	I/II	BI-505 in conjunction with ASCT in patients with MM	Terminated due to high risk of cardiopulmonary events	-
Pasireotide (SOM230 LAR)	Small somatostatin (SST) analog, interaction with PI3K/MAPK pathway, RANKL, and IGF-1	NCT04603872	II	SOM230 LAR in combination with bortezomib and dexamethasone in RRMM patients	Withdrawn before participants enrolled in the trial.	-
Sorafenib	Small molecules anti-VEGFR	NCT00536575	I/II	sorafenib and bortezomib in RRMM patients	Terminated	15% ORR, lower than preplanned
Tremelimumab	MAb, anti-CTLA-4	NCT02716805	I	Tremelimumab, durvalumab, and HDC-ASCT in TE patients with MM	Early termination due to safety signals in other studies investigating combination regimens including similar drugs	-
Durvalumab	MAb, anti-PD-L1					
Durvalumab	MAb, anti-PD-L1	NCT02685826	I/II	Durvalumab in combination with lenalidomide with and without dexamethasone in adults with NDMM	Completed	Not available
Nivolumab	MAb anti PD-1	NCT01592370 [174]	1I/II	Nivolumab as monotherapy or in combination with ipilimumab/lirilumab vs. daratumumab plus pomalidomide and dexamethasone in RRMM patients with ≥2 prior lines of therapy	Completed	Nivolumab monotherapy ORR: 4% (1/27)
Nivolumab	MAb anti-PD-1	NCT02726581	III	Nivolumab plus pomalidomide and dexamethasone or pomalidomide and dexamethasone or nivolumab plus elotuzumab, pomalidomide, and dexamethasone in RRMM patients with ≥2 prior lines of therapy	Completed	Not available
Pembrolizumab	MAb anti-PD-1	KEYNOTE-023 [175], NCT02036502	I	Pembrolizumab in combination with lenalidomide and low-dose dexamethasone in RRMM patients	Completed	ORR: 44% (22/50)
Pembrolizumab	MAb anti-PD-1	KEYNOTE-185 [146], NCT02579863	III	Lenalidomide and dexamethasone plus pembrolizumab vs. lenalidomide and dexamethasone alone	Completed	ORR: 64% (96/151)
Pembrolizumab	MAb anti-PD-1	HP-00061522 [176] NCT02289222	II	Pembrolizumab plus pomalidomide and dexamethasone in RRMM patients	Completed	ORR: 60% (29/48)
Pembrolizumab	MAb anti-PD-1	KEYNOTE-183 [145] NCT02576977	III	Pomalidomide plus dexamethasone and pembrolizumab in RRMM patients	Completed	ORR: 34% (43/125)
Atezolizumab	MAb, anti-PD-L1	NCT02431208	I	Atezolizumab alone or in combination with an IMiD (pomalidomide/lenalidomide) and/or daratumumab in MM patients	Completed	Not available
Lirilumab (BMS-986015)	MAb, anti-KIR	NCT02252263	I	Elotuzumab in combination with either lirilumab (BMS-986015) or urelumab (BMS-663513) in MM patients	Completed	Not available
Urelumab (BMS-663513)	MAb, anti-CD137					
IPH2101	MAb, anti-KIR	KIRIMID [177] NCT01217203	I	IPH2101 and lenalidomide in RRMM patients	Completed	ORR: 33% (5/15)
Relatlimab	MAb, anti-LAG-3	NCT04150965	I/II	Immuno-oncology drugs elotuzumab, anti-LAG-3, and anti-TIGIT in RRMM patients	Recruiting, ongoing	Not available
BMS-986207	MAb, anti-TIGIT					
EOS-448	MAb, anti-TIGIT	NCT05289492	I/II	EOS884448 alone and in combination with iberdomide with or without dexamethasone in RRMM patients	Recruiting, ongoing	Not available
TTI-621	Fusion protein consisting of the N-terminal domain of human SIRPα linked to a human IgG1 Fc region and of a CD47-blocking innate immune checkpoint	NCT02663518	Ia/Ib	TTI-621 in patients with RR hematologic malignancies (including MM) and selected solid tumors	Active, not recruiting	Not available
TTI-622	Fusion protein consisting of the N-terminal domain of human SIRPα linked to a human IgG1 Fc region and of a CD47-blocking innate immune checkpoint	NCT03530683	Ia/Ib	TTI-622 in patients with RRMM, RR lymphoma, and ND acute myeloid leukemia (AML)	Recruiting, not active	Not available
SRF231	MAb, anti-CD47	NCT03512340	I/Ib	SRF231 in patients with advanced solid and hematologic cancers (including MM)	Completed	Not Available
AO-176	MAb inhibiting the CD47-SIRPα checkpoint	NCT04445701	I/II	AO-176 as monotherapy and in combination with bortezomib-dexamethasone in RRMM patients	Active, not recruiting	Not available

**Abbreviations.** BM, bone marrow; ME, microenvironment; mAb, monoclonal antibody; RANKL, receptor activator of nuclear factor κ B; IGF-1, insulin-like growth factor 1; VEGFR: vascular endothelial growth factor receptor; CTLA-4, T lymphocyte-associated antigen-4; PD-L1, programmed cell death ligand-1; LAG-3, lymphocyte activation gene-3; TIGIT, T-cell immunoglobulin and ITIM domain; SIRPα, signal-regulatory protein alpha; IgG1, human immunoglobulin G1; ASCT, autologous stem-cell transplantation; MM, multiple myeloma; RR, relapsed-refractory; HDC, high-dose chemotherapy; TE, transplant-eligible; ND, newly diagnosed; ORR, overall response rate; IMiD, immunomodulatory drug.
